# Physics-informed Deep Learning for Dual-Energy Computed Tomography Image Processing

**DOI:** 10.1038/s41598-019-54176-0

**Published:** 2019-11-27

**Authors:** Maarten G. Poirot, Rick H. J. Bergmans, Bart R. Thomson, Florine C. Jolink, Sarah J. Moum, Ramon G. Gonzalez, Michael H. Lev, Can Ozan Tan, Rajiv Gupta

**Affiliations:** 1 Department of Radiology, Massachusetts General Hospital, Boston, MA USA; 2 Technical Medicine, University of Twente, Enschede, The Netherlands; 3 Harvard Medical School, Boston, MA USA; 4 Cerebrovascular Research Laboratory, Spaulding Rehabilitation Hospital, Boston, MA USA

**Keywords:** Computational science, Computed tomography

## Abstract

Dual-energy CT (DECT) was introduced to address the inability of conventional single-energy computed tomography (SECT) to distinguish materials with similar absorbances but different elemental compositions. However, material decomposition algorithms based purely on the physics of the underlying attenuation process have several limitations, leading to low signal-to-noise ratio (SNR) in the derived material-specific images. To overcome these, we trained a convolutional neural network (CNN) to develop a framework to reconstruct non-contrast SECT images from DECT scans. We show that the traditional physics-based decomposition algorithms do not bring to bear the full information content of the image data. A CNN that leverages the underlying physics of the DECT image generation process as well as the anatomic information gleaned via training with actual images can generate higher fidelity processed DECT images.

## Introduction

In conventional, single-energy computed tomography (SECT), a single X-ray tube emits a polychromatic beam that passes through the tissue to be captured by a detector array. Such projection data acquired from multiple angular directions is then reconstructed into tomographic images that encode the photon attenuation of the tissue encountered by the X-ray beam. A drawback of SECT, recognized in many studies, is its inability to distinguish materials with similar absorbances but different elemental compositions (e.g. calcium, hemorrhage, or iodine), as they are represented by similar intensity values in the CT image^1–6^. This may present a problem during radiologic interpretation when two materials with the same or similar Hounsfield Units (HU) could be present in a given anatomic location. For example, it is difficult to distinguish hemorrhage from dilute iodinated contrast when the density is in the 30–100 HU range^3,7–10^. Dual-energy CT (DECT), introduced in the mid-2000s, overcomes this problem.

DECT takes advantage of the fact that X-ray absorption is predominantly a result of Compton scattering and photoelectric effect^6^. While the former is only weakly dependent on X-ray photon energy, the latter strongly depends on it – particularly at the X-ray photon energies used in medical imaging and for materials with high atomic numbers^6^. Both of these attenuation processes are affected by the X-ray energy level used for imaging and the atomic composition of the voxel being imaged. Therefore, acquisitions at two different energy levels may be used to determine the photoelectric and Compton scattering components of the attenuation. This information is then used to characterize materials and distinguish tissues with the same or similar attenuations at SECT.

In practice, low- and high-energy DECT scans – usually 80–100 kVp and 140–150 kVp respectively – are acquired and combined to produce a simulated single-energy image that is comparable to SECT^1,3^. Both low- and high-energy DECT scans utilize a low radiation dose, so the combined scan dose is similar to or slightly greater than a conventional SECT scan. Using vendor specific post-processing software, one can apply a 2- or 3-material decomposition algorithm to the low- and high-energy images using the targeted materials’ mass-attenuation coefficients and atomic numbers^5,6,11,12^ to obtain material-specific images that represent pre-selected materials. For example, using such an algorithm, one can “subtract” out the iodine from a contrast-enhanced DECT scan to create both an iodine image and a “virtual non-contrast” (VNC) image^13^.

However, a material decomposition algorithm that is based purely on the physics of the underlying attenuation process has several fundamental limitations. Based on the assumption that each voxel is comprised of a linear combination of pre-selected target materials (e.g., iodine and water), a physics-based decomposition algorithm splits the overall dose in order to generate the two material specific images. The decomposition process, therefore, results in decreased signal-to-noise ratio (SNR) in the derived material-specific images as compared to conventional unenhanced SECT images^13^. For example, after virtual subtraction of iodine, a virtual non-contrast image has much inferior SNR as compared to a true non-contrast image which, in turn, may reduce diagnostic performance of virtual non-contrast images^14^.

Another limitation of the conventional decomposition process – informed purely by the physics of photoelectric effect and Compton scattering – is that it ignores the anatomic context and location of a voxel when processing low and high-energy slices. The elemental composition of a voxel, and consequently its intensity value on the low and high-energy images, is strongly constrained by its anatomic location. For example, a voxel located in the bone will have a very different pair of intensity values on low- and high-energy images as compared to a voxel located in the gray matter. Such location-specific information is not available to a purely physics-based decomposition technique. In theory, incorporating this information could improve the quality of the images derived from the DECT decomposition process. As a final point, the manual post-processing of standard material decomposition precludes batch processing and renders large-scale applications cumbersome and prone to human error^15–17^.

In order to overcome these disadvantages of conventional material decomposition algorithms used in practice, and to incorporate anatomy-specific information in the DECT material decomposition process, we trained a convolutional neural network (CNN) to develop a fully automated framework for DECT image processing. The training was supervised by True Non-Contrast (TNC) images acquired immediately prior to a dual-energy contrast-enhanced CT scan. The trained CNN was then used to generate predicted non-contrast head CT images, dubbed Deep Non-Contrast (DNC) images, from the DECT images. The same DECT dataset was also used to generate Virtual Non-Contrast (VNC) images using vendor-provided standard physics-based decomposition algorithm. The three sets of images – namely, the TNC, VNC, and DNC images – were quantitatively and qualitatively compared.

## Results

**Figure 1:**
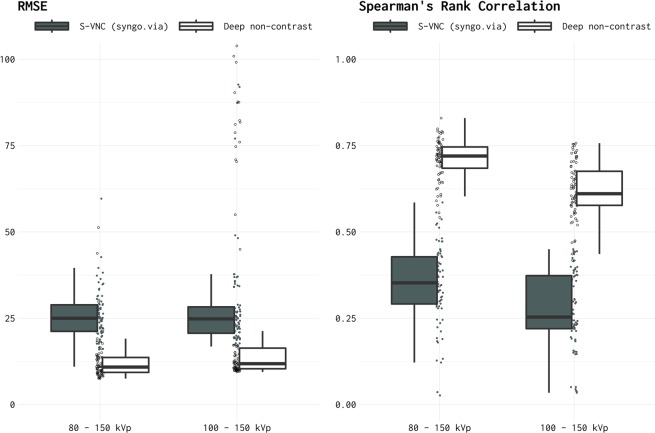
RMSE and Spearman’s Rank Correlation between TNC images versus S-VNC images reconstructed using a standard material decomposition algorithm and predicted DNC images generated using the proposed CNN. RMSE and correlations were assessed on the independent test sets. In addition to the summary box plots, RMSE and rank-correlation values for each individual image are shown as filled (S-VNC), and empty (DNC) circles.

The relative fidelity of the DNC and S-VNC images vis-à-vis the TNC images, as measured by the root mean square error (RSME) and Spearman’s rank correlation coefficient, is shown in Fig. 1.

On the independent test sets, The DNC images had RMSE = 12.8 ± 1.61 (mean ± 95% C.I.) for Data Set 1 and 25.6 ± 6.24 for Data Set 2. By comparison, the S-VNC images generated by physics-based material decomposition algorithm had an RMSE = 25.9 ± 1.63 and 28.6 ± 3.33 for Data Sets 1 and 2, respectively. Therefore, as measured by the RMSE error, the predicted DNC images demonstrated significantly higher similarity to the TNC images (i.e., significantly lower RMSE error) in the test datasets. A 2-way ANOVA (Data Set x image type – S-VNC vs. predicted DNC) showed *p* < 0.01 for the image type, without a significant main effect of Data Set or interaction effects. Thus, predicted DNC images were significantly more similar to TNC images regardless of the Data Set. RMSE outliers in the predicted DNC images in the Data Set 2 group (Fig. 1, left panel) were due to slight misalignment between DECT and TNC images, and thus between DNC and TNC images, leading to a comparison of skull voxels to brain voxels.

The Spearman’s rank correlation coefficient, encoding the rank orderings of relative pixel intensities and the correspondence between TNC and predicted DNC images, were ρ = 0.71 ± 0.02 and ρ = 0.62 ± 0.02 for the Data Sets 1 and 2, respectively. These coefficients were significantly higher than that between TNC and S-VNC images generated by the standard material composition algorithm (ρ = 0.35 ± 0.03 and 0.28 ± 0.02 for Data Sets 1 and 2, respectively). A 2-way ANOVA yielded *p* < 0.01 for main effects of Data Set and image type (S-VNC vs. predicted DNC) without any significant interaction (*p* = 0.48).

## Discussion

Since the advent of DECT, various data processing techniques have been employed to expand applications and improve diagnostic quality of this novel imaging modality. These techniques include material decomposition, generation of virtual monochromatic images for improved soft tissue contrast and differentiation (e.g., in the assessment of bone and cartilage invasion by tumor), and metal-artifact reduction^12^. While these processing techniques increase the versatility of DECT, they all suffer from the same problem: inherently low SNR and poor overall image quality of the post-processed data sets. Moreover, data post-processing is manually performed and as a result, time-consuming and prone to human error^17^.

The key contribution of our work is to show that the traditional physics-based decomposition algorithms universally used for DECT post-processing do not bring to bear the full information content of the image data. We demonstrate that a deep convolutional neural network that leverages the underlying physics of the dual-energy image generation process and the anatomic information gleaned via training with actual ground-truth images can generate higher fidelity imaging as compared with that reconstructed via traditional material decomposition algorithms. The system can function as an independent platform for processing DECT post-processing. The predicted non-contrast images (i.e., DNC images) show a substantially higher correlation with the true non-contrast (i.e., TNC) images than standard physics-based virtual non-contrast images. They have higher image quality, lower mean square error, and suffer from less information loss when compared to imaging produced from conventional material decomposition algorithms. We found no systemic relation between error from the predicted DNC and TNC images and anatomy or pathology.

While there are a number of state-of-the-art material decomposition algorithms, we had to rely only on physics-based virtual non-contrast images constructed using Syngo.Via. This was necessitated by the fact that processing raw dual-energy (as opposed to single-energy) CT data requires the knowledge of many proprietary aspects of a CT scanner such as the two X-ray spectra, detailed engineering specifications of the beam filters used, and many other relevant details that are intrinsic to the scanner. However, once the network is trained, it becomes a vendor independent platform for processing DECT images. This is one of the strengths of our approach.

**Figure 2:**
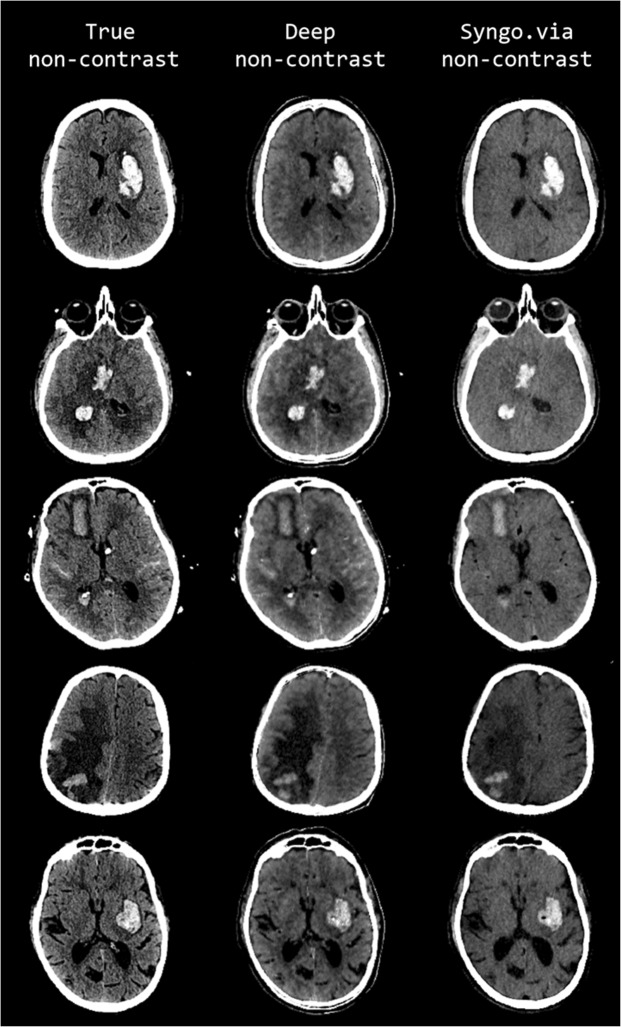
Comparison of representative examples of predicted DNC images generated using the proposed CNN (middle column, display window (40, 80)), TNC images (left column (display window (40, 80)), and S-VNC images reconstructed using a conventional material decomposition algorithm (right panel, display window (20, 60)) in the evaluation of hemorrhagic stroke. Reduced SNR on the S-VNC images erroneously de-emphasizes areas of acute hemorrhage. In comparison, the predicted DNC images demonstrate increased conspicuity of normal brain anatomy and various intracranial pathologies, including vasogenic edema, hemorrhagic infarction and subarachnoid hemorrhage. Moreover, the predicted DNC images have less noise than the TNC images, improving the visibility of subtle intracranial hemorrhage. First row: 49-year-old male presenting with unilateral weakness and aphasia secondary to acute ICH within the left basal ganglia. Vasogenic edema surrounding the ICH is more easily identified on the DNC image than the S-VNC image. Second row: 47-year-old unresponsive female with a large acute ICH in the right basal ganglia and intraventricular hemorrhagic extension. There is trans-ependymal CSF flow, consistent with acute hydrocephalus as a result of intraventricular hemorrhagic extension, which is well-visualized on the predicted DNC and TNC images and not well seen on the S-VNC image. Third row: 72-year-old female with acute subarachnoid hemorrhage and acute ICH secondary to a ruptured anterior communicating artery aneurysm. The TNC and predicted DNC images depict multifocal acute subarachnoid hemorrhage and trans-ependymal CSF flow from acute hydrocephalus, which are not identifiable on the S-VNC image. Note that the differentiation and extent of acute subarachnoid hemorrhage is improved on the predicted DNC image compared to the TNC image, most notable along the anterior left frontal lobe. Fourth row: 64-year-old male presenting with altered mental status secondary to a large right parietal ICH, which is better evaluated in extent on the predicted DNC image compared to the S-VNC image. The extent of surrounding vasogenic edema is also better characterized on TNC and predicted DNC images. Slight posterior morphological differences between the TNC and other images are secondary to registration inaccuracy. Fifth row: 80-year-old male presenting with acute-onset right facial droop, right-sided weakness and expressive aphasia secondary to an acute ICH involving the left basal ganglia and left insula. Both TNC and predicted DNC images better delineate normal anatomy and the extent of vasogenic edema surrounding the ICH. Moreover, the predicted DNC image demonstrates reduced noise in comparison to the TNC image, allowing for better gray-white matter differentiation.

**Figure 3:**
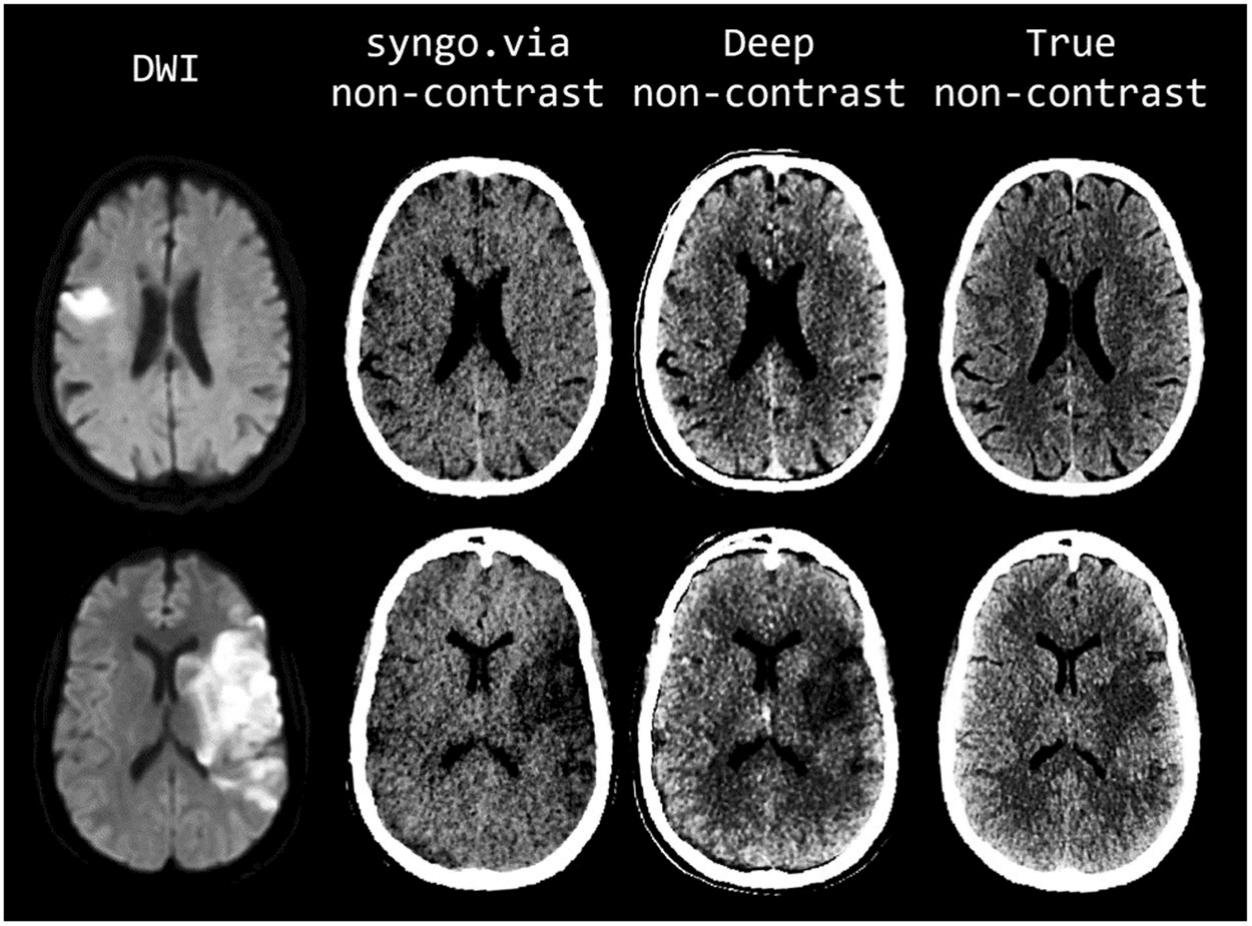
Comparison of representative examples of diffusion-weighted images (first column), S-VNC images reconstructed using a conventional material decomposition algorithm (second column, display window of (20, 60)), predicted DNC images generated using the proposed CNN (third column, display window (40, 80)), and TNC images (fourth column, display window (40, 80)) in the evaluation of acute ischemic stroke. Decreased SNR on the VNC images results in poor delineation of true infarct size and location. In contrast, the improved image quality of the predicted DNC images allows for better visualization of normal brain anatomy and pathology, as confirmed by DWI. First row: 75-year-old male with acute-onset left facial droop and left arm weakness secondary to an acute ischemic infarct within the anterior right middle cerebral artery territory. Loss of gray-white matter differentiation and local sulcal effacement secondary to the infarct are best visualized on the predicted DNC image in comparison to the VNC and TNC images. Of note, identification of the infarct is particularly difficult on the VNC image. Second row: 51-year-old unresponsive female who presented with a large acute left middle cerebral artery territory infarction. The true size and location of the acute infarct, as confirmed by DWI imaging, is most conspicuously identified on the predicted DNC image secondary to improved SNR.

The improved image quality of DNC imaging in comparison to standard VNC imaging is visually apparent by qualitative assessment of both hemorrhagic and ischemic strokes in our results. Representative cases of hemorrhagic and ischemic strokes are shown, respectively, in Figs. 2 and 3. Upon evaluation of each column in these cases, the predicted DNC images demonstrate the increased conspicuity, and in some cases more complete characterization, of various intracranial pathologies, including intraparenchymal hemorrhage, subarachnoid hemorrhage, and parenchymal vasogenic edema. There is also improved anatomic definition on DNC images with enhanced gray-white matter differentiation within the brain parenchyma when compared to the VNC images. These observations further support the diagnostic utility of our generated images for not only the visualization of brain anatomy but also brain pathology.

Our results are consistent with, and supplement a prior study showing that convolutional networks can provide excellent performance in noise suppression on DECT images. Zhang *et al*^18^. reported a remarkable, up to 95%, reduction in noise standard deviation in tissue, bone, and mixture regions on a digital phantom reconstructed from dual-energy projections. Since the primary target of this work was noise reduction, Zhang *et al*. used the mean and SD of the noise level in selected region of interest (ROI). While this is an excellent method to assess performance when the ground truth is known (in this case, digital phantom and projection data), it is not directly applicable to the current study, where clinical images (not phantoms) at two energy-levels (and not the projection data) are used to train the network. Therefore, a direct comparison of performance to this prior work is not immediately possible.

Our approach relied on global measures of performance because the overall image quality is important in routine day-to-day clinical practice. For example, when evaluating intracerebral hematoma for evidence of expansion at DECT, it is important to have a high-fidelity representation of both the virtual non-contrast image and the iodine overlay image. Our images show a marked improvement in the generated virtual non-contrast images that rivals true non-contrast images. While the intracranial contents of the processed images were superior, the proposed framework resulted in extracranial artifacts on some images. Given the scope and focus of the current study, and the fact that these artifacts were readily identified, their presence was deemed clinically unimportant. Thus, this novel, physics-informed deep learning framework represents a superior approach over the traditional material decomposition process, both on objective (i.e. reduced error rate) and subjective (i.e. visual inspection) scales.

Our results indicate that the visibility of anatomy and pathology on the predicted DNC images may even surpass TNC images because of increased SNR, as judged by qualitative inspection (Figs. 2 and 3). For example, our model’s increased sensitivity for subtle intracranial hemorrhage would be valuable in selecting patients with acute infarction for tissue plasminogen activator (tPA) therapy. In addition to more sensitive detection of intracranial hemorrhage, our results also demonstrate improved visibility of acute ischemic stroke. The DNC imaging displayed in Fig. 3 more accurately depicts the true size and location of acute ischemic strokes in comparison to TNC imaging, as confirmed by DWI imaging.

Prior work has shown that deep learning networks (e.g., RED-CNN, wavelet residual networks, generative adversarial networks), can provide significant improvements in image quality of conventional (i.e. single-energy) CT images^19–22^. These studies have demonstrated that approaches based on deep convolutional networks can offer significant advantages by allowing images to be acquired with lower doses of radiation. However, it should be noted that our approach is applicable to reconstruction of images from the raw DECT data. It does not represent a classical noise reduction technique. A direct comparison with classical noise reduction algorithms is therefore not possible.

In addition to their potential for improved image quality, deep learning pipelines allow for accelerated workflow by automatically generating individual DNC images. By removing the human intervention in DECT post-processing, the fully automated character of the proposed framework will enable more efficient imaging throughput and make material decomposition less prone to human error. In clinical practice, faster image post-processing allows for more efficient radiologic interpretation and reporting and decreases overall examination turnaround times. Rapid image availability can also better guide clinical decision-making and patient management in acute clinical scenarios. Similarly, in the research setting, automated post-processing facilitates larger-scale image production and utilization. This method is completely generalizable on other DECT paradigms such as kVp-switching, twin-beam, and dual-layer scanners. In fact, truly multispectral photon counting detectors are even more promising for this approach as they provide perfectly registered images in multiple energy bins.

## Outlook

The generation of high-quality, non-contrast DNC images may obviate the need for separately obtaining non-contrast images in situations where they are routinely acquired in conjunction with contrast-enhanced images, thereby reducing the overall radiation dose to individuals. When coupled with photon counting detectors, where multi-spectral data will be available to the CNN to perform multi-level coincident material decompositions, image quality and material decomposition may be further improved. However, *in vivo* validation with a wider range of patients and pathologies will first be required to assess our model’s generalizability.

## Methods

### Patient population

This retrospective study was approved by the Partners HealthCare Institutional Review Board (protocols #2010P001506 and #2015P000607) and conducted in accordance with HIPAA guidelines. Since the data was acquired as part of the routine clinical care and analyzed retrospectively, requirement for informed consent was waived by the IRB. The authors had control of the data and information presented in this manuscript. Data used in the study are available from the corresponding author by request.

This study included scans of patients who were referred for dual-energy head CT between November 2014 and August 2018 for evaluation of hemorrhagic or ischemic stroke. All scans were performed using the standard departmental protocol at our institution, which includes a non-contrast head CT followed by a contrast-enhanced Dual-Energy head CT. The inclusion criteria were availability of an artifact-free conventional unenhanced non-contrast, single-energy CT scan prior to contrast administration as well as low and high-energy components of a contrast-enhanced dual-energy CT scan performed immediately afterwards.

Scans from 209 adult patients were included. This cohort was comprised of 107 patients with intracranial hemorrhage and 102 with acute ischemic stroke. Twenty-one patients were excluded because either one or both dual-energy components of the scan were not saved in our Picture Archiving and Communication System (PACS) or were unreadable. Six additional individuals were excluded as their DECT scans were obtained at energy levels that did not follow the standard departmental protocol. Thus, the final cohort comprised 182 patients, provided 37,611 two-dimensional (512 × 512) image slices for training, validation, and testing of the convolutional neural network (described below in detail).

### Data acquisition

All DECT images were obtained using two dual source CT scanners (Somatom Definition Force™ or Flash™, Siemens Healthineers, Erlangen, Germany) and post-processed using the vendor provided analysis platform (syngo.via, Siemens Healthineers, Erlangen, Germany). This proprietary software is provided by the vendor, and employs engineering specifications of the beam filters used, the X-ray spectrum and other scanner intrinsic details. The two X-ray sources were set at the tube voltages of 80 kVp and 150 kVp-Sn for Flash™ (n = 78 patients) and 100 kVp and 150 kVp-Sn for Force™ (n = 104 patients). The designation “Sn” in the high-energy component of the DECT scan denotes that a tin filter was used to increase the spectral separation between the high- and low-energy spectra. The difference in scanners (and scan protocol) approximately at the midpoint of our study is due to the change of our Emergency Department dual-energy CT from Somatom Flash™ to Somatom Force™ scanner.

The following scan protocol was used: tube A at 80 or 100 kVp; tube B, with tin filter, at 150 kVp; effective tube currents of 714 and 168 mA for tubes A and B, respectively; and a collimation of 14 × 1.2 mm. The effective dose of a dual-energy CT scan was comparable to that of a conventional CT scan (~3 mSv per scan). Each image stack consisted of a low- and high-kVp series, both with a voxel resolution of 1 mm^3^.

Of the 37,611 image slices that were analyzed, 13,635 were obtained as 80 kVp - 150 kVp-Sn energy pairs (Data Set 1) and the remaining 23,976 were obtained as 100 kVp - 150 kVp-Sn energy pairs (Data Set 2). All image slices were partitioned into training, validation, and independent test data sets in the following proportions: 74.5%, 14.5%, and 11.0% for Data Set 1, and 74.4%, 15.0%, and 10.6% for the Data Set 2, such that images from a given patient were kept within only one data set.

### Image Pre-processing

The image data for each patient consisted of an unenhanced “True” Non-Contrast (TNC) single-energy scan, followed by a contrast-enhanced dual-energy scan of the brain. The dual-energy scans were post-processed using syngo.via (Syngo Dual Energy Brain Hemorrhage, Siemens Healthcare) to obtain three-material decomposition from the low- and high-kVp images. The decomposition was performed using water and hemorrhage as the base materials, and any measured deviations from the linear combination of the attenuations of the two base materials were attributed to the third material, in this case, the contrast material (iodine). The post-processing algorithm used an iodine ratio of 2.12, slice thickness of 1 mm, and all other parameters set to their default values in order to generate the virtual non-contrast (hereafter referred to as S-VNC image). All images were reconstructed at 1 mm slice thickness, with a 0.6 mm slice spacing, and an average field-of-view of 228 mm (range: 197–285 mm).

For direct comparison, the S-VNC and TNC scans were co-registered to a standard space and re-sliced using the SPM12 toolbox (v.7219; Functional Imaging Laboratory, Welcome Trust Centre for Neuroimaging, Institute of Neurology at University College London, UK) for MATLAB 2018a (MathWorks, Inc., Natick, Massachusetts, US). After co-registration, skull-stripping was performed using a validated automatic implementation of the FSL 6.0 Brain Extraction Tool with 3D Gaussian smoothing using *σ = *1 mm^3^ kernel size^23,24^. Skull-stripping was supervised by two of the authors (R.H.U.B. and M.G.P.) to guard against software failures and possible elimination of brain parenchyma adjacent to the inner table of the skull. To further minimize artifacts at the boundary secondary to co-registration and skull-stripping, the final brain mask was eroded using a spherical 5-pixel × 5-pixel spherical structuring element and cropped.

### Generation of deep non-contrast images

#### Physics-informed Lookup VNC (L-VNC) Generation

**Figure 4:**
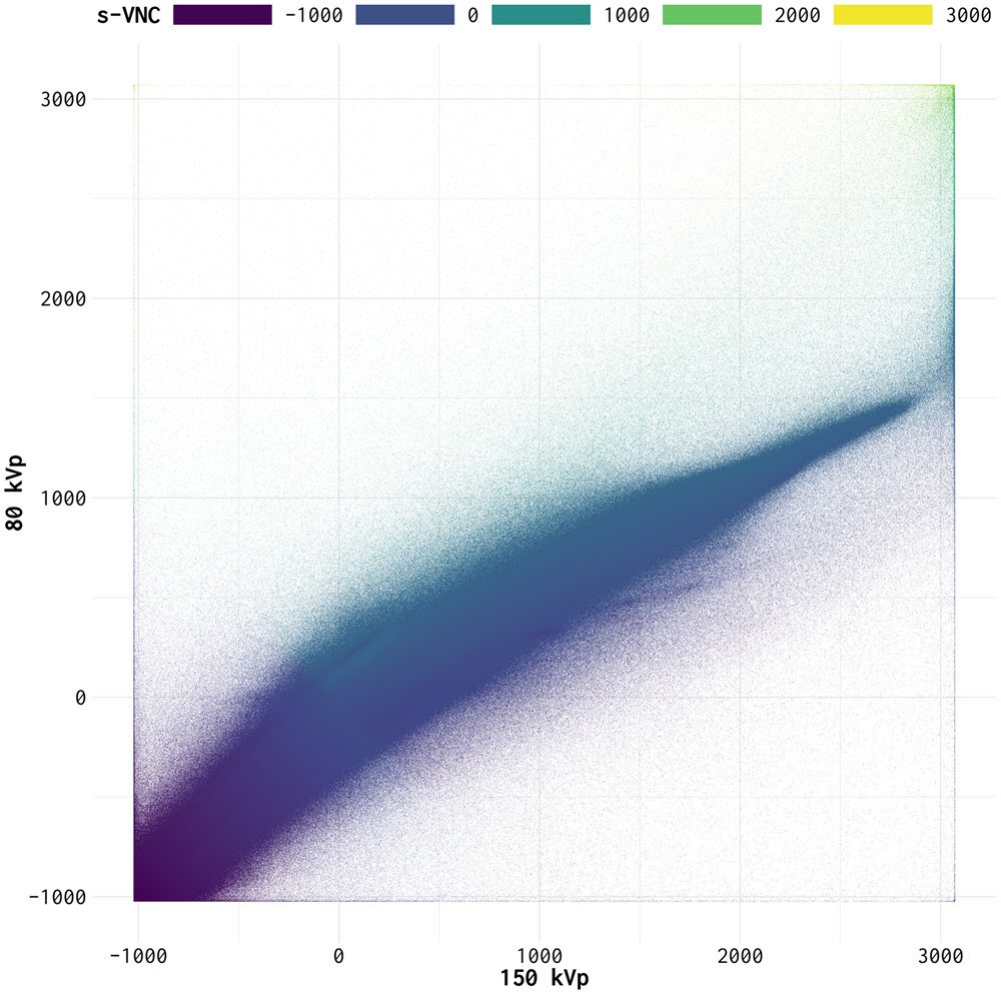
Raster plot of the relationship between images at different DECT energies and S-VNC images constructed using a material decomposition algorithm (syngo.via, Siemens Healthineers, Erlangen, Germany). The x- and y-axes show pixel values for low- and high-energy images, respectively. The value at a specific x-y location indicates the corresponding pixel value on S-VNC images.

By design, S-VNC captures the physics of the underlying material decomposition process. It describes how pairs of low- and high-energy value pairs are combined to provide an intensity that represents contrast-free attenuation. Subsequently, the mapping between S-VNC intensities and low- and high-energy pairs was used to create a 2-dimensional “lookup image” (Fig. 4). Any non-mapped points were imputed using an iterative inter/extrapolation method based on a penalized least squares method^25,26^ for 100 iterations. Any remaining missing input-output relations were filled with zeros. The mapping encoded in the lookup image was then applied to generate lookup-VNC images (hereafter referred as to L-VNC image) for all DECT low- and high-energy image pairs in the training set. L-VNCs were median filtered using a 5-pixel × 5-pixel kernel to minimize noise.

#### Network architecture

**Figure 5:**
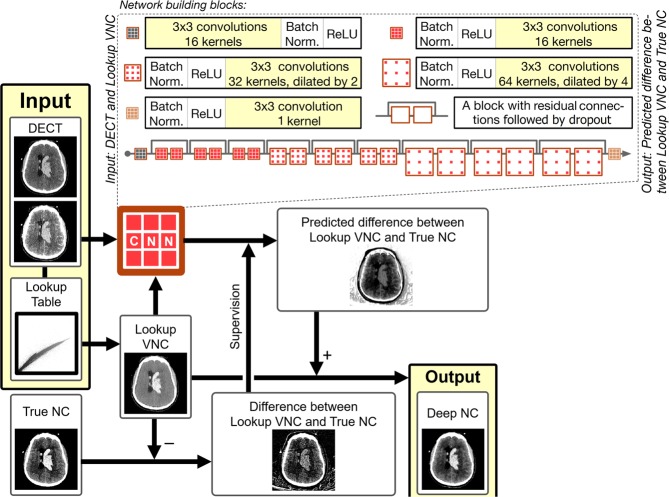
A high-level representation of the analysis framework.

To correct the error between the previously described L-VNC and TNC we developed and trained a supervised 2-dimentional convolutional neural network (CNN) based on the ResNet architecture^27^. The ResNet architecture allows for increased network depth; reducing overfitting and allowing increased abstraction while retaining relative trainability. The network consists of three sets of each three residual blocks, with increasing dilation factor of 1, 2, and 4 (see Fig. 5 inset). Dilation factor of deeper layers increases to account for features of different scales. Residual connections ensure the learning of residual mappings instead of ‘plain’ mappings. In deeper networks, it is easier to optimize weights of these residual mappings. Network depth was established empirically, as increased depth proved to be too hard to train, and a shallow network reduced performance substantially. Further architectural details are described elsewhere in the literature^27,28^.

The network was implemented in Python 3.6.5 using Keras 2.2.3 as the high-level API and TensorFlow 1.12.0 as the backend. Training was done on a Nvidia GeForce GTX 1080 Ti graphics processing unit (GPU). The training batch size was eight, and images were down sampled to 256 × 256 due to memory constraints. Adam optimization with L2 regularization, default weight initialization, and a constant learning rate of 10^−5^ was used for training. An L2 regularization value of 0.02 was used during weight optimization, with a dropout of 0.5. Performance analysis on the training and validation sets was limited to the voxel constituting to the brain parenchyma using a brain mask, to prioritize performance enhancement in this area. The error, i.e. performance, was computed by the root mean squared error between these two sets of included voxels. Two models with the same CNN architecture – one for the Data Set 1 and the other for the Data Set 2 – were trained separately. This entailed generating different lookup images, L-VNC images, and DECT image pair inputs for the CNN models.

The input to the network were three sets of input images, namely, the low-energy (80 or 100 kVp) and high-energy (150 kVp-Sn) DECT image pair and the corresponding L-VNC image. The training was supervised by *the difference* between the TNC images and the generated L-VNC image. Consequently, the output of the trained network generated the expected difference between true non-contrast (TNC) and L-VNC images. Therefore, adding this predicted difference to L-VNC image should yield a predicted non-contrast CT image that approximates the TNC image. We labeled these generated images as Deep Non-Contrast or DNC images (see Fig. 5). Models were trained until the validation loss function reached a plateau. It took 30 epochs spanning 9 hours for validation loss to plateau at 5.65 HU for Data Set 1, and 33 epochs (14 hours) at 19.12 HU for Data Set 2.

### Performance assessment

Absolute values of pixel intensities (in Hounsfield Units) of the predicted DNC images were compared with the TNC images using root mean squared error (RMSE). Similar analysis was applied to S-VNC vs TNC images. From a practical, radiologic perspective, relative rank ordering of pixel intensities between different images is more important than their absolute values. For example, while factors such as window and level may change the overall pixel intensities, as would adding a DC offset to an image, the relative order of pixel values is not changed by these operations. To assess the fidelity with which the relative order of pixel intensities is replicated in the DNC and S-VNC images as compared to the TNC images, we also used Spearman’s rank-order correlation to quantify similarity between original and predicted images. Both performance measures were calculated for batches of 32 randomly selected images to stratify for number of brain voxels in each performance measurement. All statistical analyses were performed using R Language for Statistical Computing (version 3.5.1).
